# Supplementary material for: Guava Leaf Extract Exhibits Antimicrobial Activity in Extensively Drug-Resistant (XDR) Acinetobacter baumannii
Source: Molecules. 2024 Dec 28;30(1):70. doi: 10.3390/molecules30010070 (PMC11722033; doi:10.3390/molecules30010070)
Supplement: Supplementary file 1 [file molecules-30-00070-s001.zip › molecules-3337934-supplementary.pdf]

molecules

MDPI

Supplementary material

Figure S1. Determination of the minimum inhibitory concentration (MIC) of different clinical isolates of *Acinetobacter baumannii*.

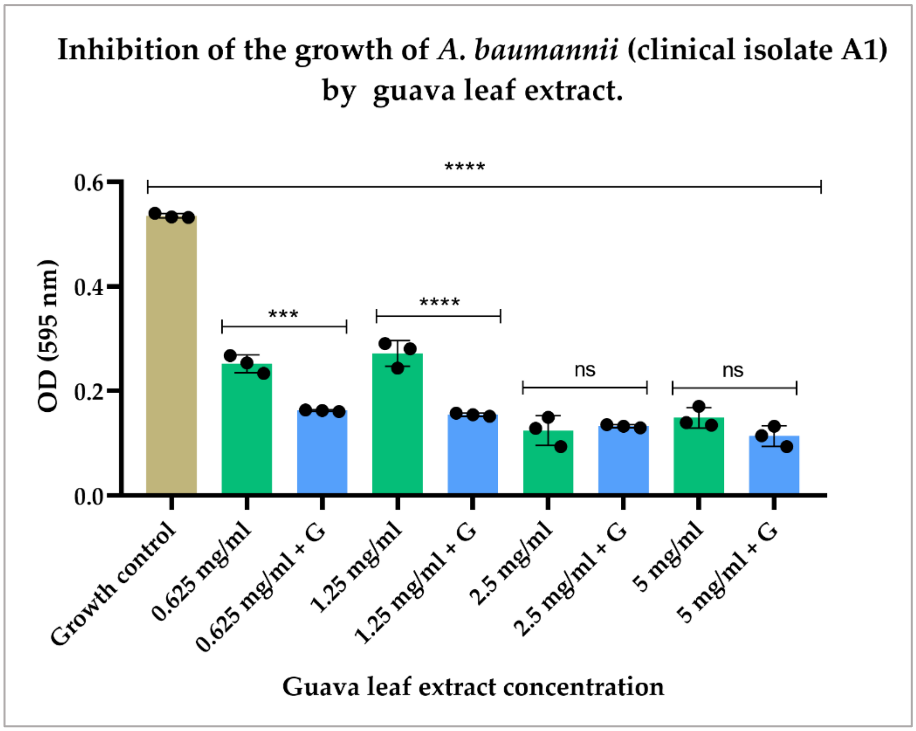

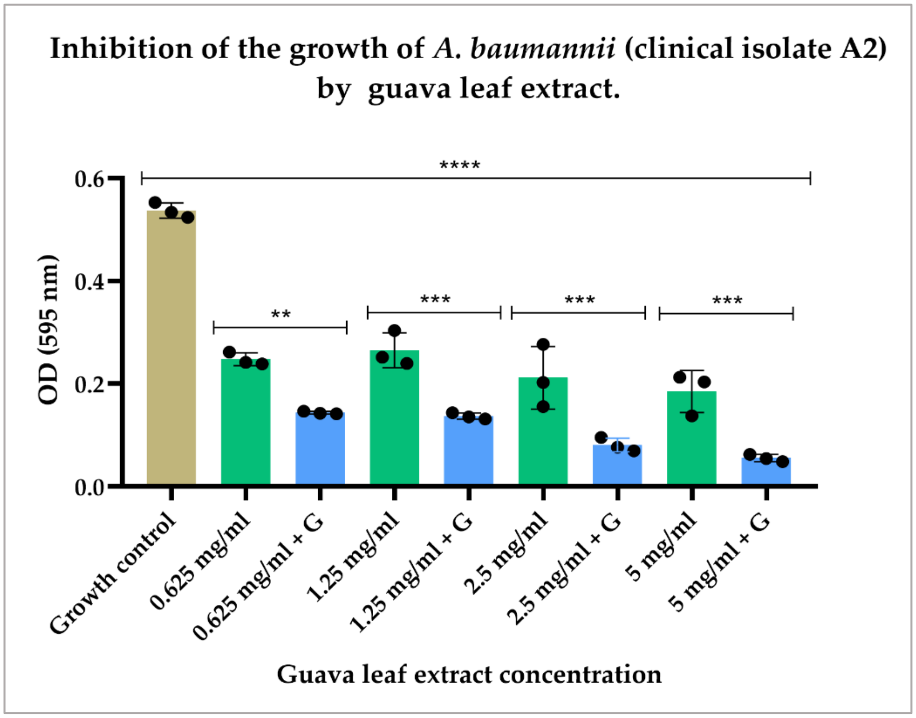

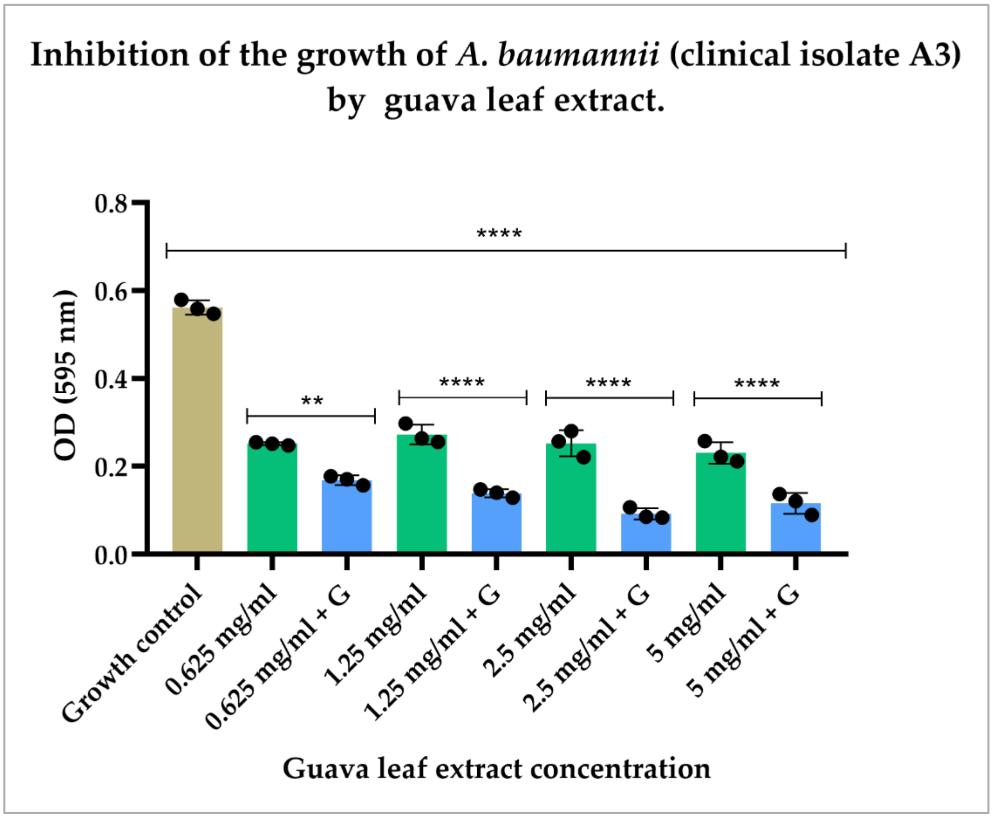
Inhibition of the growth of A. baumannii (clinical isolate A3) by guava leaf extract.
OD (595 nm)
0.8
0.6
0.4
0.2
0.0
****
**
****
****
****
Growth control
0.625 mg/ml
0.625 mg/ml + G
1.25 mg/ml
1.25 mg/ml + G
2.5 mg/ml
2.5 mg/ml + G
5 mg/ml
5 mg/ml + G
Guava leaf extract concentration

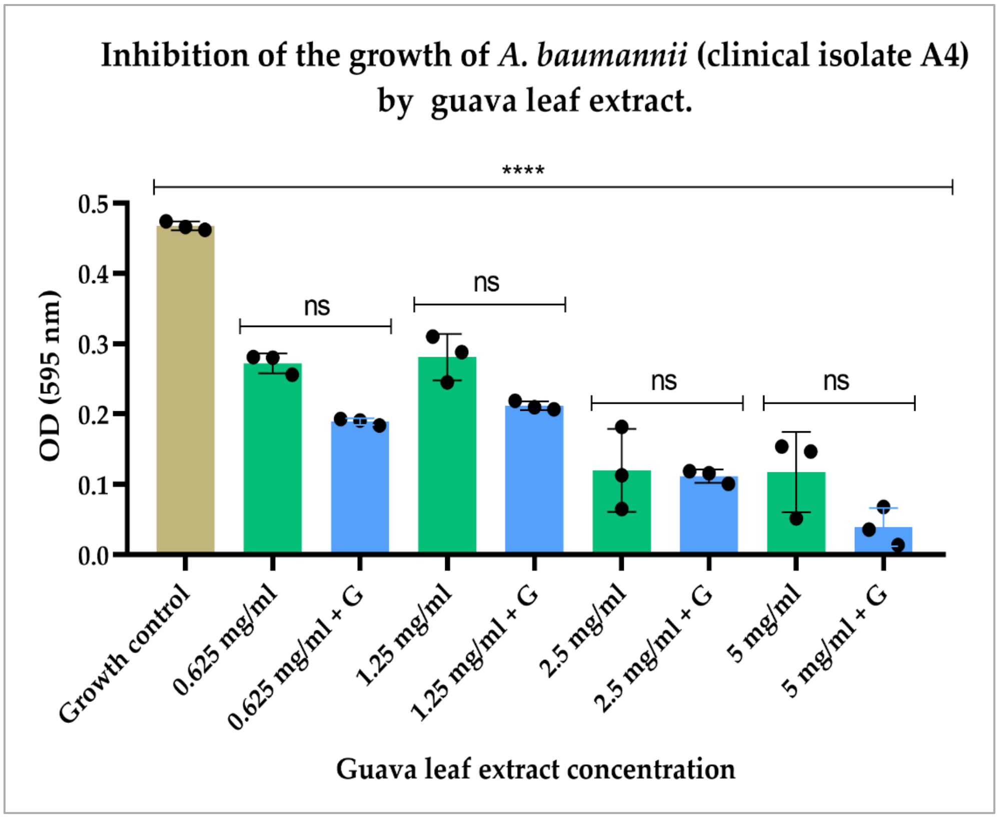
Inhibition of the growth of A. baumannii (clinical isolate A4) by guava leaf extract.
OD (595 nm)
0.5
0.4
0.3
0.2
0.1
0.0
****
ns
ns
ns
ns
Growth control
0.625 mg/ml
0.625 mg/ml + G
1.25 mg/ml
1.25 mg/ml + G
2.5 mg/ml
2.5 mg/ml + G
5 mg/ml
5 mg/ml + G
Guava leaf extract concentration

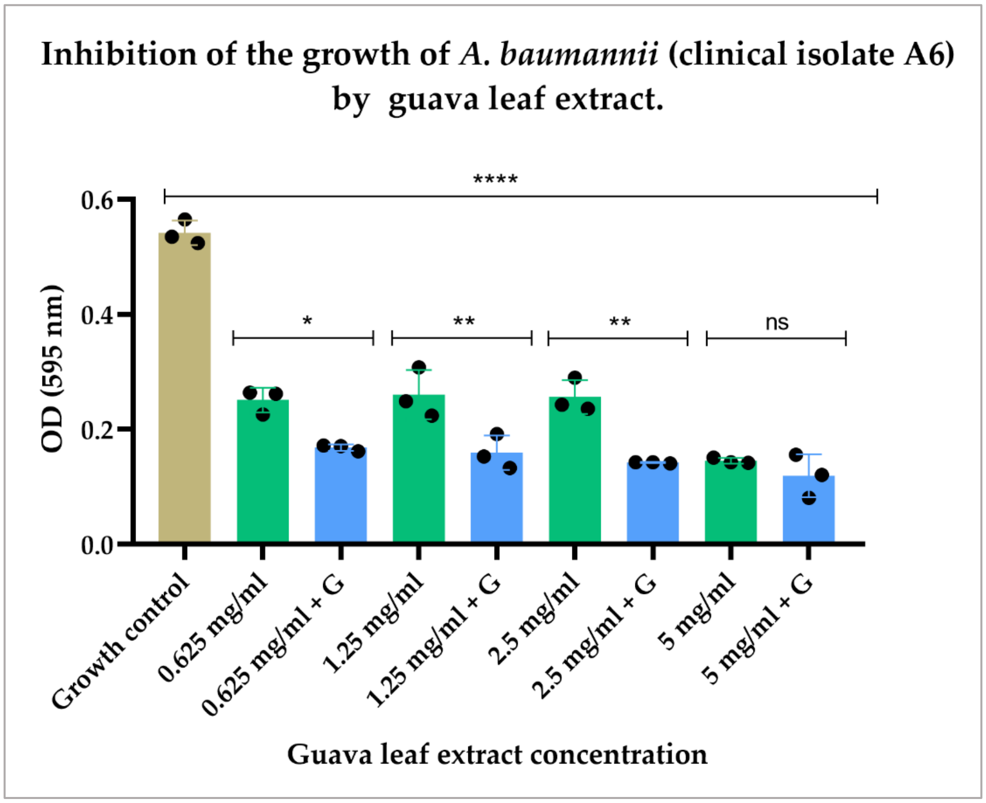
Inhibition of the growth of A. baumannii (clinical isolate A6) by guava leaf extract.
****
*
**
**
ns
0.6
0.4
0.2
0.0
OD (595 nm)
Growth control
0.625 mg/ml
0.625 mg/ml + G
1.25 mg/ml
1.25 mg/ml + G
2.5 mg/ml
2.5 mg/ml + G
5 mg/ml
5 mg/ml + G
Guava leaf extract concentration

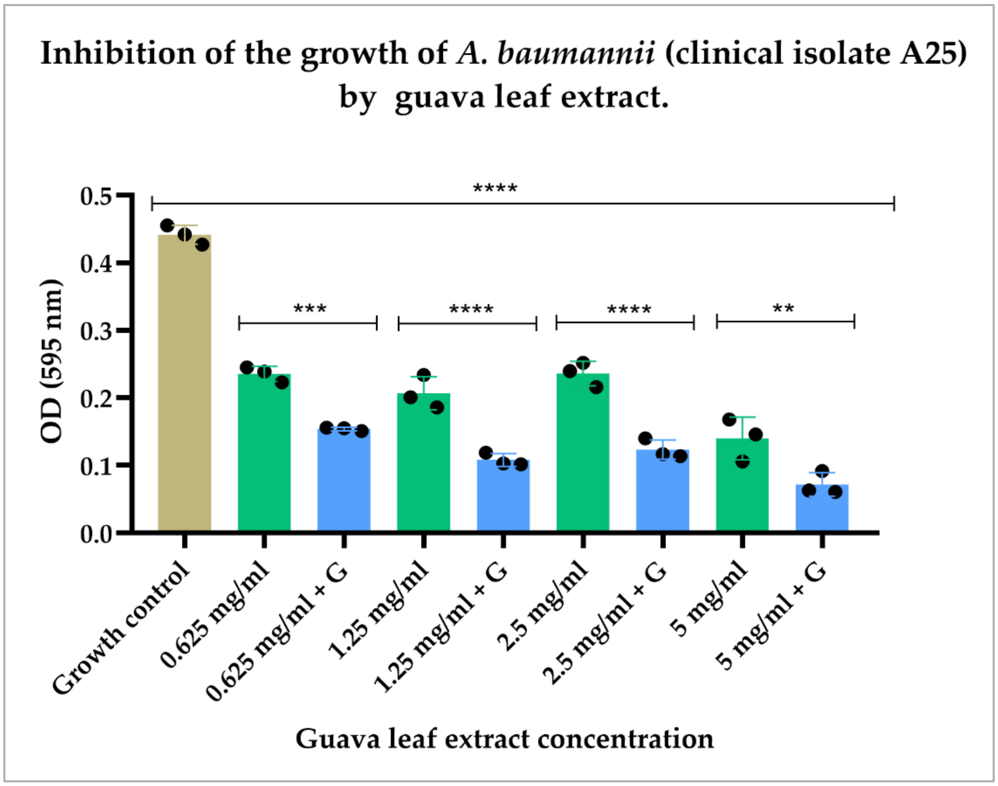
Inhibition of the growth of A. baumannii (clinical isolate A25) by guava leaf extract.
****
***
****
****
**
0.5
0.4
0.3
0.2
0.1
0.0
OD (595 nm)
Growth control
0.625 mg/ml
0.625 mg/ml + G
1.25 mg/ml
1.25 mg/ml + G
2.5 mg/ml
2.5 mg/ml + G
5 mg/ml
5 mg/ml + G
Guava leaf extract concentration

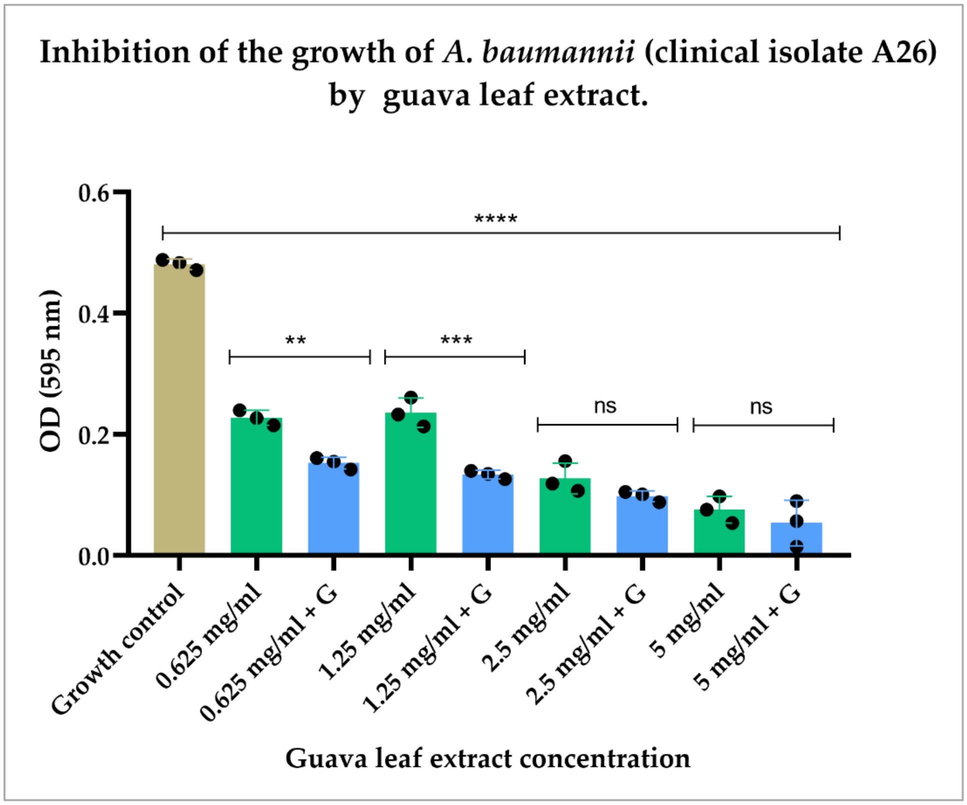
Inhibition of the growth of *A. baumannii* (clinical isolate A26) by guava leaf extract.
OD (595 nm)
0.0
0.2
0.4
0.6
****
**
***
ns
ns
Growth control
0.625 mg/ml
0.625 mg/ml + G
1.25 mg/ml
1.25 mg/ml + G
2.5 mg/ml
2.5 mg/ml + G
5 mg/ml
5 mg/ml + G
Guava leaf extract concentration

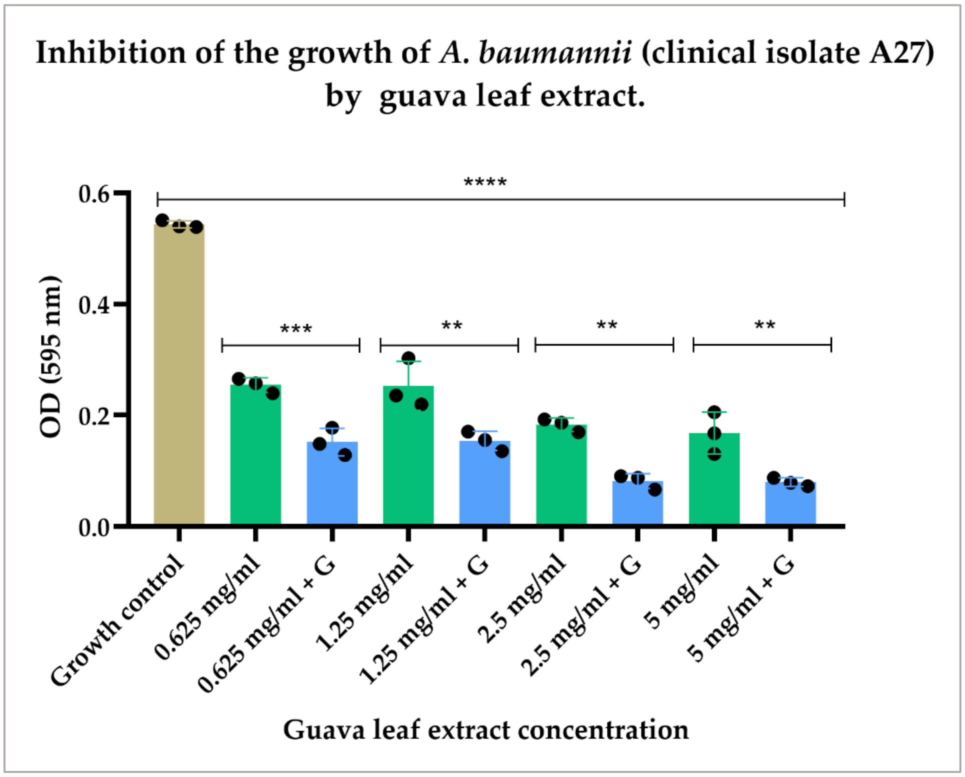
Inhibition of the growth of *A. baumannii* (clinical isolate A27) by guava leaf extract.
OD (595 nm)
0.0
0.2
0.4
0.6
****
***
**
**
**
Growth control
0.625 mg/ml
0.625 mg/ml + G
1.25 mg/ml
1.25 mg/ml + G
2.5 mg/ml
2.5 mg/ml + G
5 mg/ml
5 mg/ml + G
Guava leaf extract concentration

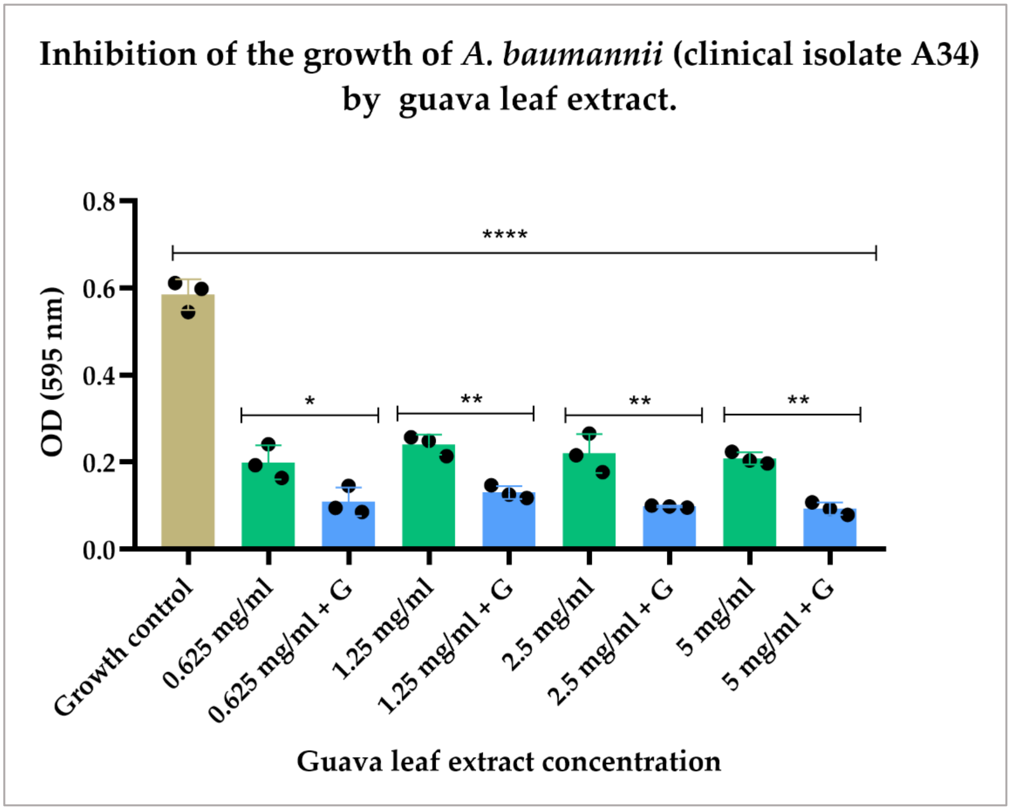
Inhibition of the growth of A. baumannii (clinical isolate A34) by guava leaf extract.
OD (595 nm)
0.8
0.6
0.4
0.2
0.0
****
*
**
**
**
Growth control
0.625 mg/ml
0.625 mg/ml + G
1.25 mg/ml
1.25 mg/ml + G
2.5 mg/ml
2.5 mg/ml + G
5 mg/ml
5 mg/ml + G
Guava leaf extract concentration

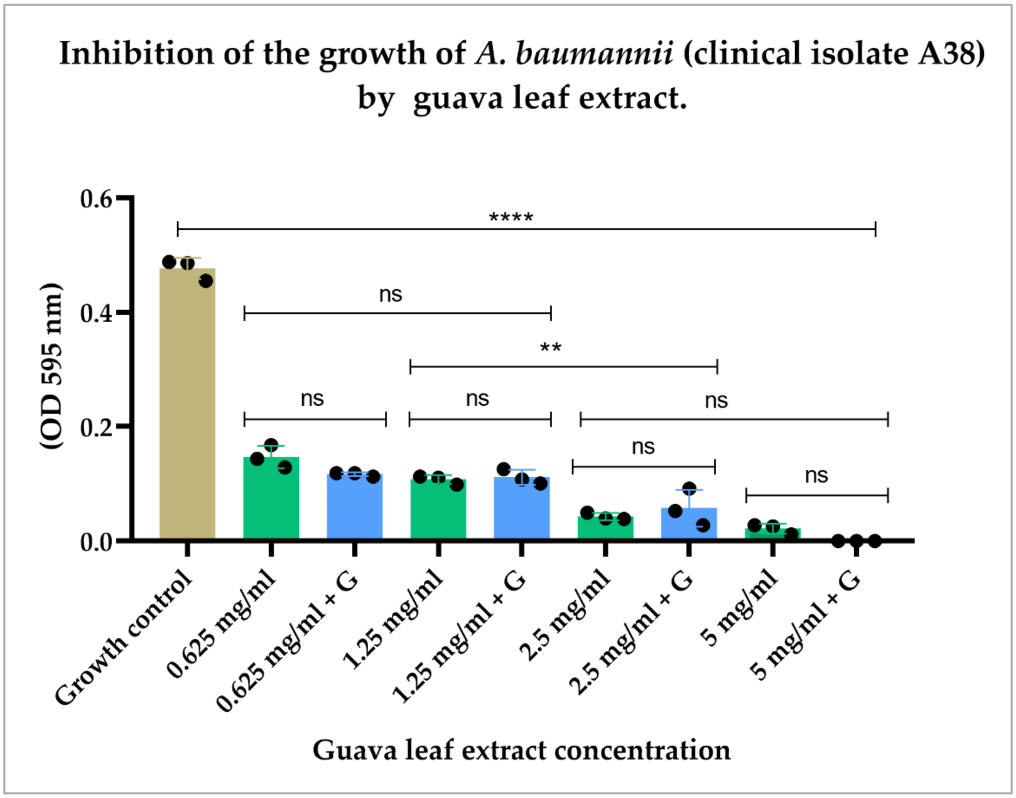
Inhibition of the growth of A. baumannii (clinical isolate A38) by guava leaf extract.
(OD 595 nm)
0.6
0.4
0.2
0.0
****
ns
**
ns
ns
ns
ns
ns
Growth control
0.625 mg/ml
0.625 mg/ml + G
1.25 mg/ml
1.25 mg/ml + G
2.5 mg/ml
2.5 mg/ml + G
5 mg/ml
5 mg/ml + G
Guava leaf extract concentration

Table S1. Antimicrobial susceptibility patterns of *Acinetobacter baumannii* clinical isolates evaluated.

Antibiotic classes	Antbiotic	Antimicrobial susceptibility patterns									
		A1	A2	A3	A4	A6	A25	A26	A27	A34	A38
Beta-lactams	Ampicillin	R	R	R	R	R	R	R	R	R	R
	Dicloxacillin	R	R	R	R	R	R	R	R	R	R
	Carbanicillin	R	R	R	R	R	R	R	R	R	R
Penicillins/sulfonamides	Ampicillin/ Sulbactam	R	R	R	R	R	R	R	R	R	R
Sulfonamides	Trimethoprim/ sulfamethoxazole	R	R	R	R	R	R	R	R	R	R
Cephalosporins	Cefepime	R	R	R	R	R	R	R	R	R	R
	Ceftazidime	R	R	R	R	R	R	R	R	R	R
	Ceftriaxone	R	R	R	R	R	R	R	R	R	R
	Cephalotin	R	R	R	R	R	R	R	R	R	R
	Cefotaxime	R	R	R	R	R	R	R	R	R	R
Carbapenems	Doripenem	R	R	R	R	R	R	R	R	R	R
	Imipenem	R	R	R	R	R	R	R	R	R	R
	Meropenem	R	R	R	R	R	R	R	R	R	R
Quinolones	Ciprofloxacin	R	R	R	R	R	R	R	R	R	R
	Norfloxacin	R	R	R	R	R	R	R	R	R	R
Nitrofurantoin	Nitrofurantoin	R	R	R	R	R	R	R	R	R	R
Aminoglycosides	Gentamicin	R	R	R	R	R	R	R	R	R	R
	Amikacin	R	R	R	R	R	R	R	R	R	R
	Netilmicin	R	R	R	R	R	R	R	R	R	R
Tetracycline	Tetracycline	R	R	R	R	R	R	R	R	R	R
Penicillins/beta-lactamase inhibitors	Piperacillin/ Tazobactam	R	R	R	R	R	R	R	R	R	R
Cephalosporin/beta-lactamase inhibitor	Ceftazidime/avibactam	R	R	R	R	R	R	R	R	R	R
Polimixin E	Colistin	S	S	I	I	I	I	I	I	I	I
Glycylcycline	Tigecycline	R	I	S	I	I	S	S	S	S	S
Macrolides	Erythromycin	R	R	R	R	R	R	R	R	R	R
Glycopeptides	Vancomycin	R	R	R	R	R	R	R	R	R	R

** R = resistant, I = intermediate resistant, S = susceptible.
